# Resveratrol Attenuates Liver Inflammation in Non-Alcoholic Fatty Liver Disease by Activating PINK1-Mediated Mitophagy

**DOI:** 10.3390/ani16071022

**Published:** 2026-03-27

**Authors:** Shujing Tan, Ran Yu, Longwei Sun, Manman Shen, Juan Framirez Pedroso, Osmani Chacón Chacón, Chengmin Li, Weiguo Zhao

**Affiliations:** 1 Jiangsu Key Laboratory of Sericultural and Animal Biotechnology, School of Biotechnology, Jiangsu University of Science and Technology, Zhenjiang 212100, China; 231111801109@stu.just.edu.cn (S.T.); 18912250570@163.com (R.Y.); sunlongwei163@163.com (L.S.); shenman2005@163.com (M.S.); 2 Key Laboratory of Silkworm and Mulberry Genetic Improvement, Ministry of Agriculture and Rural Affairs, The Sericultural Research Institute, Chinese Academy of Agricultural Sciences, Zhenjiang 212100, China; 3 Research Center on Protein Plants and Bionatural Products, No. 902, 7th Avenue, Playa, La Habana 11300, Cuba; jpedroso@bionaturasm.cu (J.F.P.); osmanichacon75@gmail.com (O.C.C.)

**Keywords:** fatty liver disease, transition dairy cows, resveratrol, mitophagy, PINK1, NLRP3 inflammasome

## Abstract

Resveratrol (RES) has been shown to exhibit therapeutic efficacy against fatty liver disease. Yet, the molecular mechanisms by which RES ameliorates liver injury remain unclear. The aim of this study was to investigate the therapeutic effect and mechanism of resveratrol in fatty liver disease. It was found that dairy cows with fatty liver exhibit characteristic hepatic pathologies, including ballooning degeneration, lipid accumulation and elevated serum AST and ALT levels. Parallel to these changes, we observed significant upregulation of the NLRP3 inflammasome alongside suppression of mitophagy in the liver. Additionally, it was demonstrated in vitro that resveratrol pretreatment effectively alleviated PA-triggered NLRP3 inflammasome activation and mitochondrial dysfunction. Furthermore, RES’s mitigating effects against NLRP3 inflammation and mitochondrial injury were reversed by suppressing PINK1-medicated mitophagy. In vivo experiments further demonstrated that resveratrol administration attenuated HFD-induced liver injury and lipid accumulation in a mouse model, concurrent with suppressed NLRP3 activation and an increase in mitophagy, further confirming the mechanism identified in vitro. Our findings reveal that RES ameliorates fatty liver injury primarily by inhibiting the NLRP3 inflammasome through PINK1-mediated mitophagy, which provides a potential novel therapeutic strategy for mitigating fatty liver disease.

## Simple Summary

Our research indicates that resveratrol improves fatty liver disease by enhancing mitophagy, coupled with inhibiting NLRP3-driven inflammation. These findings not only provide new molecular insights into the pathogenesis of bovine fatty liver, but also pave the way for resveratrol-based interventions, and provide a feasible and active strategy to control the disease in dairy cows in the transitional period.

## 1. Introduction

Dairy cows commonly enter a state of energy deficit during the perinatal period, owing to reduced feed consumption and increased metabolic energy needs. This metabolic state will cause adipose tissue to mobilize substantial non-esterified fatty acids (NEFAs). Excess fatty acids are mainly taken up by the liver, which leads to the excessive accumulation of triacylglycerol (TG), which eventually induces fatty liver [1,2]. Fatty liver is a significant metabolic disorder in transition dairy cows. And this condition impacts the overall health as well as the productive and reproductive performance of cows, ultimately posing a severe threat to dairy farming [3,4,5].

Previous animal and cellular studies have demonstrated that fatty liver in periparturient dairy cows is usually accompanied by an inflammatory reaction [6,7]. NOD-like receptor protein 3 (NLRP3) is a key congenital immune polyprotein assembly which can be triggered by high concentrations of NEFA. Its activation drives the inflammatory response by orchestrating the processing of Caspase-1 together with the production and release of IL-1β and IL-18 [8]. More importantly, activation and assembly of the NLRP3 inflammasome stimulate an increase in pro-inflammatory cytokines, resulting in the amplification of liver inflammation and associated hepatocyte injury [7,9,10]. Therefore, identifying therapeutic approaches aimed at the NLRP3 inflammasome cascade is crucial for ameliorating inflammatory responses in the livers of cows suffering from fatty liver.

NLRP3 activation leads to the recruitment of autophagy components LC3 and p62 to compromised mitochondria, triggering the PINK1-guided mitophagy pathway [11,12]. As a specialized mechanism, mitophagy plays a critical role in sustaining mitochondrial homeostasis via the clearance of defective mitochondria. New evidence shows that mitophagy can inhibit NLRP3 inflammatory signaling conduction, thus weakening the inflammatory response [13]. It is worth noting that it has been proven that mitophagy in cows with fatty liver disease is impaired, and increased NEFA levels can inhibit mitophagy activity in calf liver cells by reducing the colocation of LC3, PINK1 and mitochondria [14,15,16]. Therefore, promoting mitophagy to block the activation of NLRP3 inflammatory bodies might constitute a viable strategy for treating inflammatory diseases in cows with fatty liver.

Resveratrol (RES, 3,5,4′-trihydroxystilbene), is a bioactive natural polyphenol derived from plant sources like grapes, peanuts, and berries [17]. Numerous studies have confirmed the hepatoprotective action of this bioactive compound molecule in cellular and animal models, primarily mediated through its antioxidative, anti-inflammatory properties [18,19,20,21]. More importantly, resveratrol has been found to modulate inflammatory injury through the activation of PINK1-dependent mitophagy and suppression of NLRP3 inflammasome assembly [22,23,24]. These findings led us to hypothesize that resveratrol mitigates inflammation in bovine fatty liver disease via enhanced mitophagy. Accordingly, this research aimed to elucidate the protective mechanisms of RES in regulating the inflammation response across experimental models of fatty liver. This investigation may offer insights into novel treatment strategies for fatty liver during the transition period.

## 2. Materials and Methods

### 2.1. Reagents

The following primary antibodies were used: anti-PINK1 (1:2000; Proteintech, Wuhan, China, 81991-4-RR), anti-p62 (1:5000; Proteintech, 18420-1-AP), anti-Parkin (1:1000; Proteintech, 14060-1-AP), anti-LC3 (1:2000; Proteintech, 14600-1-AP), and anti-NLRP3 (1:2000; Proteintech, 30109-1-AP). Anti-Caspase1 (1:2000; Wanleibio, Shenyang, China, WL03450) and anti-IL-1β (1:2000; Santa Cruz Biotechnology, Santa Cruz, CA, USA, sc-12742) antibodies were also employed. Secondary detection utilized an anti-rabbit IgG antibody (1:10,000; Biosharp, Hefei, China, BL004A). Resveratrol (≥99%, Jing Zhu Biotechnology, Nanjing, China, JZ23011510) was dissolved in DMSO for in vitro studies. Palmitic acid (PA, P0500, Sigma-Aldrich, Saint Louis, MO, USA) was first dissolved in 0.1 M NaOH and combined with 10% Albumin bovine serum (≥98%, A600332; Sangon Biotech, Shanghai, China) to achieve an 8 mM palmitate solution.

### 2.2. Experimental Animals

This study was conducted in compliance with institutional guidelines and was authorized by the Animal Ethics Committee of Jiangsu University of Science and Technology (Protocol G2025SJ0422). Based on hepatic triglyceride content, sixteen early-postpartum Holstein cows (within 15 days of calving) from a commercial herd were selected and equally allocated to a normal liver group (TG < 1%, *n* = 8) and a fatty liver group (TG > 5%, *n* = 8). All animals were maintained under identical housing and nutritional conditions throughout the study. On day 15 postpartum, liver tissue samples were collected from each cow via percutaneous needle biopsy using a stainless steel trocar inserted between the 11th and 12th intercostal space. Then the biopsy sample was rinsed with sterile saline, and carefully sliced to minimize mechanical damage. All samples were processed in the same manner: a portion of the liver tissue intended for histopathological evaluation was placed in 4% paraformaldehyde, then the remaining samples were rapidly frozen in liquid nitrogen and stored at −80 °C for subsequent analysis. Blood samples were collected via jugular venipuncture using Vacutainer tubes (no anticoagulant; Kangweishi, Hebei, China, China), allowed to clot for 30 min at room temperature, and centrifuged at 3500× *g* for 15 min at 4 °C. The separated serum was stored at −80 °C until analysis.

A fatty liver disease model was established using BALB/c mice (6–8 weeks, 20–25 g), procured from the Laboratory Animal Center of Jiangsu University. All animals were housed under standard laboratory conditions. Following a one-week adaptation period, the mice were acclimatized to conditions associated with one of the following five groups (*n* = 8): control (CON, basal diet), high-fat diet (HFD), and three HFD groups with different doses of RES (HFD + RES30, HFD + RES60, HFD + RES120). Throughout the 10-week experimental period, all non-control mice were maintained on a high-fat diet deriving 45% of its energy from fat. Resveratrol, suspended in 0.5% CMC-Na, was administered to the respective groups once-daily via oral gavage. Doses of 30, 60, and 120 mg/kg/day (10 mL/kg body weight volume) were selected based on previous studies [25]. Upon completion of the 10-week dietary intervention, mice were fasted for 12 h prior to euthanasia by cervical dislocation. Following orbital enucleation, blood was collected into plain microtubes without anticoagulant and centrifuged (3000× *g*, 10 min, 4 °C), and the separated serum was preserved at −80 °C. The liver was promptly excised, washed in neutral buffered saline, and dissected to remove surrounding tissue. For histopathological evaluation, a section of liver tissue was placed in 4% paraformaldehyde. Remaining samples were flash-frozen using liquid nitrogen and preserved at −80 °C pending further analysis.

### 2.3. Cell Culture and Treatments

The murine AML-12 hepatocyte line was selected for mechanistic studies of the PINK1/Parkin mitophagy pathway, owing to its well-defined phenotype and suitability for gene silencing experiments. The AML-12 hepatocyte line was obtained from the School of Biotechnology at Jiangsu University of Science and Technology. AML-12 hepatocytes were cultured using DMEM/F-12 medium (G4612, Servicebio, Wuhan, China), supplemented with 10% fetal bovine serum (B210705RP, Gibco, Grand Island, NY, USA) and 1% penicillin-streptomycin (100 U/mL, Invitrogen, Carlsbad, CA, USA). Cells were incubated in a humidified 37 °C incubator supplied with 5% CO_2_. The concentrations of PA and RES used in this study were optimized through preliminary experiments to ensure effective yet subtoxic treatment conditions [26,27]. Based on preliminary optimization experiments to assess cell viability, in the lipotoxicity induction experiments, AML-12 cells were treated with 600 μM PA for 24 h, and pretreated with 30 μM RES 24 h before PA treatment.

### 2.4. Cell Transfection

To knock down PINK1 expression in vitro, hepatocytes received transfection of PINK1-specific siRNA (siPINK1) or non-targeting siNC, obtained from GenePharma (Shanghai, China). Transfection was performed using siRNA transfection reagent (G04026, Genepharma, Shanghai, China) in accordance with the manufacturer’s instructions. The PINK1 siRNA sequence is 5′-CUGCACGUUAGCUGCAAATT-3′, the antisense sequence is 5′-UUUGCAGCUAAGCGUGCAGTT-3′, the siNC sequence is 5′-CACUCAAGAUUGUCAGCAATT-3′, and the antisense sequence is 5′-UUGCUGACAAUCUUGAGUGAG-3′.

### 2.5. Hematoxylin and Eosin (H&E) Staining

Following fixation in 4% paraformaldehyde for 24–48 h, liver samples underwent routine dehydration and paraffin embedding, and were then sectioned into 4 μm slices. The tissue sections underwent xylene dewaxing, ethanol gradient rehydration, and subsequent staining with hematoxylin–eosin (G1004; Servicebio, Wuhan, China) following conventional methods. Following staining, the sections were subsequently dehydrated, cleared, and mounted with neutral-balsam, and were then examined using an Olympus light microscope (Olympus, Tokyo, Japan) for histology. Histological assessment was carried out by two independent pathologists unaware of the experimental groups.

### 2.6. Oil Red O (ORO) Staining

For lipid droplet visualization, freshly collected liver tissues were immersed in optimal cutting temperature medium and flash-frozen. A low-temperature thermostat maintained at −18 °C was used to cut the frozen sample into slices of 8 μm thickness; then, they were fixed in 75% alcohol for 15 min at room temperature and washed thoroughly with distilled water. For staining, sections were treated with Oil Red O (G1015; Servicebio, Wuhan, China) for 15 min, and then re-dyed with Sumu essence to visualize the nucleus. Stained sections were sealed using glycerin gelatin and promptly observed with an Olympus microscope (Olympus, Tokyo, Japan) for lipid accumulation assessment. Steatosis was assessed by calculating the percentage of hepatocytes with macrovesicular lipid droplets in ten randomly selected non-overlapping fields per section at 200× magnification. The mean percentage was determined for each sample. ORO-positive areas were analyzed using ImageJ (version 1.54f) and presented as a percentage of the total field area.

### 2.7. Immunohistochemistry

Liver tissue sections were prepared from paraffin-embedded samples, which were sectioned, and then underwent stepwise clearing in xylene and graded ethanol rehydration (from 100% to 70%). Antigen retrieval was performed by heating sections in 10 mM sodium citrate buffer at 95 °C for 20 min. Endogenous peroxidase activity was blocked by treating sections with 3% H_2_O_2_ for 10 min under ambient conditions. For blocking, sections were first incubated with normal goat serum (1:10; AR0009, Boster, Wuhan, China) in PBS (45 min, room temperature). Subsequently, they were incubated with primary antibodies (diluted in PBS with 5% goat serum) overnight at 4 °C.

For negative controls, the procedure was identical except that no primary antibody was applied. Subsequent to PBS washing, sections were probed by HRP-linked secondary antibodies (1:500) under room temperature for 45 min. Visualization was achieved using 3,3′-diaminobenzidine substrates followed by counterstaining with Mayer’s hematoxylin. All images were captured with an Olympus fluorescence microscope (Olympus, Tokyo, Japan).

### 2.8. Biochemical Assays

Plasma levels of total superoxide dismutase (T-SOD) and glutathione peroxidase (GSH-Px) activity, as well as malondialdehyde (MDA) content, were assayed with specific commercial kits (Nanjing Jiancheng Bioengineering Institute, Nanjing, China), following the supplier’s guidelines. Absorbance was measured at wavelengths of 560 nm (T-SOD), 420 nm (GSH-Px), and 532 nm (MDA), employing a microplate reader. The results for enzyme activity are presented as U/mg prot; MDA content is shown as nmol/mg prot.

Serum activity of alanine aminotransferase (ALT) and aspartate aminotransferase (AST) was determined with commercial assay kits (Nanjing Jiancheng Bioengineering Institute). Measurements were conducted at 505 nm and 510 nm, respectively, with results expressed as U/g prot for tissues and U/L for serum. For hepatic triglyceride (TG) quantification, liver tissue was accurately weighed and homogenized in ice-cold physiological saline (0.9%) at a ratio of 1:9 (weight/volume). The homogenate was centrifuged at 2000× *g* for 10 min at 4 °C, and the supernatant was collected. TG content was measured using a commercial kit (Nanjing Jiancheng Bioengineering Institute) based on the GPO-PAP method, with absorbance read at 500 nm. Total cholesterol (TC) was assayed using the COD-PAP method following the same kit instructions. Concentrations were expressed as mmol/g protein for tissue samples and mmol/L for serum.

### 2.9. Enzyme-Linked Immunosorbent Assay (ELISA)

Commercial ELISA kits (Nanjing BioAssay Biotechnology, Nanjing, China) were employed to determine serum levels of IL-1β, TNF-α, and IL-6, as instructed by the supplier. The minimum detectable concentrations were as follows: IL-1β < 2.344 pg/mL (BYHS500007), TNF-α < 1.563 pg/mL (BYHS500006), and IL-6R < 15.625 pg/mL (BYHS101006). The measurable ranges were 9.375–300 pg/mL, 6.25–200 pg/mL, and 62.5–2000 pg/mL. The measurable intervals for the three analytes were 9.375–300, 6.25–200, and 62.5–2000 pg/mL. OD readings were obtained with a BioTek microplate reader (Washington, DC, USA). ELISA was performed in duplicate with readings taken at 450 nm. The intra-assay CV was below 10%, and the inter-assay CV under 15%. Validation was performed via a standard curve included on each plate. These accuracy parameters are consistent with the manufacturer’s specifications (CV in measurement < 10%; CV in measurement < 15%).

### 2.10. Measurement of Mitochondrial ROS Concentration (mtROS)

To quantify mitochondrial reactive oxygen species (mtROS), MitoSOX Red (GC68230, GlpBio Technology, San Diego, CA, USA) was utilized as per the supplier’s instructions. In brief, AML-12 cells seeded on coverslips were exposed to 100 μL of MitoSOX Red solution (diluted in serum-free medium from the 2000× stock) and kept under light-protected conditions at ambient temperature for 15 min. Following two washes in serum-free medium, images were obtained using a Nikon Eclipse NI fluorescence microscope (Olympus, Japan). The red fluorescence signal, indicative of mitochondrial superoxide production, was acquired with excitation at 510 nm and emission at 580 nm.

### 2.11. Determination of Mitochondrial Membrane Potential (Δψm)

The assessment of mitochondrial membrane potential (Δψm) was conducted using a JC-1 Assay Kit (E-CK-A301, Elabscience, Wuhan, China). Following treatments, AML-12 cells (2 × 10^5^/well) were subjected to washing followed by incubation in JC-1 working solution by first diluting the 500× stock with the provided dilution buffer, followed by thorough mixing to prevent probe aggregation, at 37 °C for 20 min. Following repeated washes, fluorescent images were obtained using a microscope. (Olympus, Tokyo, Japan).

### 2.12. Real-Time Quantitative PCR Analysis

Total RNA was extracted from AML-12 cells and murine/bovine liver tissue samples (20 mg) using FreeZol Reagent (R711, Vazyme, Nanjing, China). A NanoDrop 1000 spectrophotometer (Thermo Scientific, Waltham, MA, USA) was employed to measure RNA concentration and assess its purity. After DNase I treatment to eliminate genomic DNA contamination, cDNA was generated via reverse transcription using PrimeScript RT Master Mix (RR036A, Takara, Tokyo, Japan). Using SYBR^®^ Premix Ex Taq™ (RR420A, Takara, Japan), qPCR was run on an Applied Biosystems 7500 HT system as per the kit protocol. See Table S1 for primer sequences. Using β-Actin for normalization, gene expression was quantified by the 2^−ΔΔCt^ method.

### 2.13. Western Blotting

Following homogenization of liver tissues and AML-12 hepatocytes, samples were centrifuged (12,000× *g*, 10 min, 4 °C) to collect protein. A BCA assay kit (P0012, Beyotime Biotechnology, Shanghai, China) was employed to measure the protein concentration of the supernatants. After separation by SDS-PAGE, proteins were electrotransferred to PVDF membranes using a semi-dry system (GenScript, Nanjing, China). For immunoblotting, membranes were incubated with primary antibodies (4 °C, overnight) specific against NLRP3, Caspase-1, IL-1β, P62, LC3-B, PINK1, Parkin, β-Actin, and Tubulin. After three TBST washes, membranes were incubated with HRP-linked secondary antibodies (1 h, RT). Enhanced chemiluminescence (Pierce, Rockford, IL, USA) was used for band visualization, and quantified using ImageJ software (National Institutes of Health, Bethesda, MD, USA). All assays were conducted in triplicate to ensure reproducibility.

### 2.14. Immunofluorescent Staining

Following fixation in 4% paraformaldehyde, cells underwent blocking using goat serum, then were sequentially incubated with a primary antibody against LC3B (1:200) and a fluorescent secondary antibody (1:1000; BL003A, Biosharp, Hefei, China). Nuclei were counterstained with DAPI (BL105A, Biosharp Life Sciences, Hefei, China). Images were captured on an Olympus fluorescence microscope (Tokyo, Japan).

### 2.15. Statistical Analysis

All experimental data represent a minimum of three biological replicates with triplicate independent repetitions. GraphPad Prism 10.1.2 (GraphPad Software, San Diego, CA, USA) was used for statistical analysis. Data normality was assessed using the Shapiro–Wilk test, and homogeneity of variance was verified by the Brown–Forsythe test to confirm assumptions for parametric testing. For comparisons between two groups, Student’s *t*-test was applied. For multi-group comparison, we used one-way variance analysis and Tukey post-test, while the two-group comparison used Student’s *t*-test. Statistical significance was established at *p* < 0.05, with *p* < 0.01 considered highly significant.

## 3. Results

### 3.1. Hepatic Pathological Characteristics in Dairy Cows with Fatty Liver

**Figure 1:**
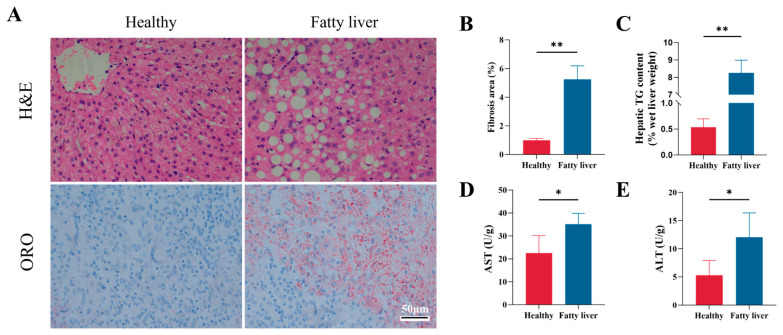
Hepatic histopathological changes and serum biochemical parameters in dairy cows with fatty liver. (**A**) Representative photomicrographs of liver sections stained with H&E and ORO. (**B**) Quantification of Oil Red O-positive areas in liver sections. (**C**) Hepatic TG content in healthy and cows with fatty liver. (**D**,**E**) Serum activity of ALT and AST in healthy and fatty liver groups. Data are presented as mean ± SEM (*n* = 8), * *p* < 0.05, ** *p* < 0.01; statistical differences were assessed by *t*-test. Scale bar, 50 μm.

H&E and ORO staining demonstrated marked ballooning degeneration and lipid accumulation in bovine fatty liver tissues (Figure 1A,B). Consistently, hepatic TG content was markedly higher in cows with fatty liver compared to healthy controls (Figure 1C). In addition, cows with fatty liver exhibited notably higher serum ALT and AST levels than their healthy counterparts (Figure 1D,E).

### 3.2. Hepatic NLRP3 Inflammasome Activation and Autophagic Impairment in Dairy Cows with Fatty Liver

**Figure 2:**
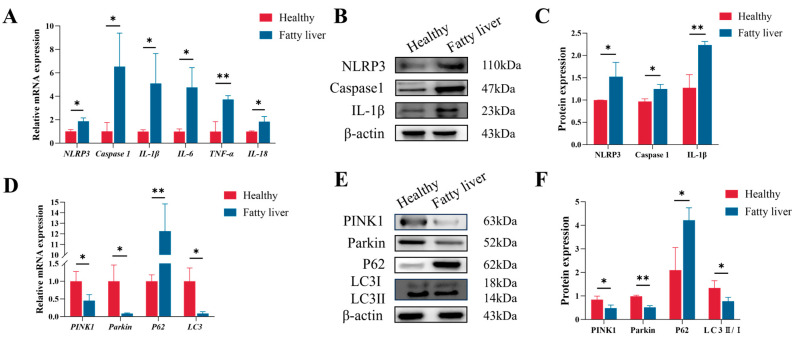
Hepatic inflammatory response and mitophagy alterations in transition dairy cows with fatty liver. (**A**) mRNA abundance of *NLRP3*, *Caspase-1*, *IL-1β*, *IL-6*, *TNF-α* and *IL-18* in the livers from different groups; (**B**,**C**) the protein abundance of NLRP3, Caspase-1 and IL-1β in the livers from different groups; (**D**) mRNA abundance of PINK1, Parkin, P62 and LC3; (**E**,**F**) the protein abundance of PINK1, Parkin, P62 and LC3 in the livers from different groups. Data are presented as mean ± SEM (*n* = 8), * *p* < 0.05, ** *p* < 0.01; statistical differences were assessed by a *t*-test.

Relative to healthy controls, those with fatty liver demonstrated significant upregulation in the mRNA levels of *NLRP3*, *Caspase-1*, *IL-1β*, *IL-6*, *TNF-α*, and *IL-18* (Figure 2A), as well as increased hepatic protein levels of NLRP3, Caspase-1, and IL-1β in the liver (Figure 2B,C). Conversely, as illustrated in Figure 2D–F, both mRNA and protein levels of PINK1 and Parkin were markedly reduced in fatty liver tissues compared to the controls. Furthermore, the ratio of LC3-II to LC3-I was reduced, while P62 protein levels were significantly elevated, indicating impaired autophagic flux in cows with fatty liver.

### 3.3. Resveratrol Attenuates NLRP3-Mediated Inflammatory Responses in PA-Induced AML-12 Cells

**Figure 3:**
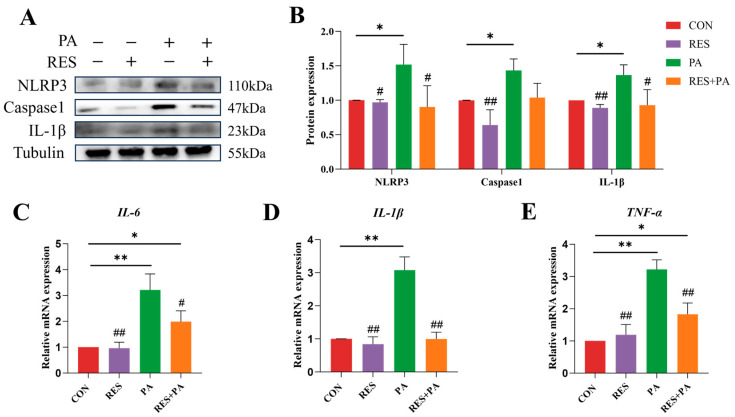
The effect of resveratrol treatment on NLRP3 inflammasome activation in AML-12 cells treated with palmitic acid. (**A**,**B**) The protein abundance of NLRP3, Caspase-1 and IL-1β in AML-12 cells from different groups. (**C**–**E**) mRNA abundance of *IL-6*, *IL-1β* and *TNF-α* in AML-12 cells from different groups. Data are presented as mean ± SEM. * *p* < 0.05, ** *p* < 0.01 vs. CON group; ^#^ *p* < 0.05, ^##^ *p* < 0.01 vs. PA group.

To investigate the anti-inflammatory effects of RES on hepatocytes under lipotoxic stress, we employed a PA-induced AML-12 cell model. Figure 3A,B demonstrate that PA treatment markedly promoted inflammatory responses in AML-12 Cells. This was supported by elevated protein levels of NLRP3, Caspase-1, and IL-1β. In addition, transcriptional upregulation of the cytokines *IL-6*, *IL-1β*, and *TNF-α* was also evident (Figure 3C–E). On the contrary, RES intervention markedly lowered the expression level of the NLRP3 inflammasome and its downstream inflammatory cytokines. Taken together, these results suggest that resveratrol intervention effectively inhibits the inflammatory signaling cascade mediated by the NLRP3 inflammasome in PA-induced AML-12 cells.

### 3.4. Resveratrol Ameliorates PA-Induced Mitochondrial Impairment in AML-12 Cells

**Figure 4:**
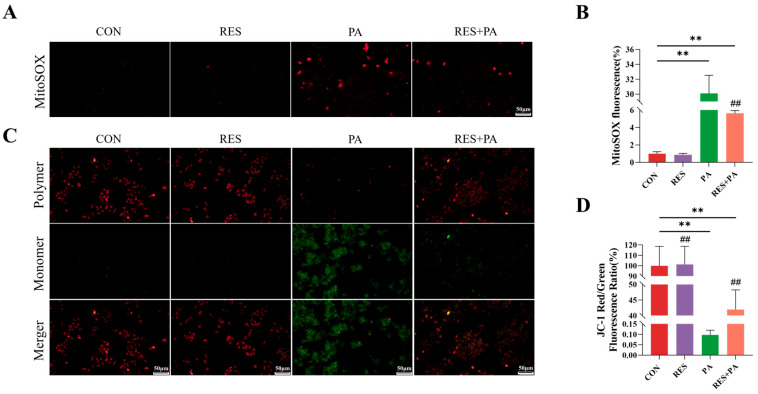
Resveratrol alleviates PA-induced mitochondrial damage in AML-12 cells. (**A**,**B**) Detection of intracellular and mitochondrial ROS. (**C**,**D**) Assessment of ΔΨm by JC-1 staining. Data are presented as mean ± SEM, ** *p* < 0.01 vs. CON group; ^##^ *p* < 0.01 vs. PA group. Scale bar, 50 μm.

In order to determine whether resveratrol reduces PA-induced mitochondrial damage, we measured the levels of mtROS and ΔΨm in AML-12 cells. The data in Figure 4 demonstrate that PA stimulation seriously damages mitochondrial function, as shown in the loss of ΔΨm and the accumulation of mtROS. On the contrary, resveratrol pretreatment effectively restores ΔΨm and inhibits the production of mtROS, proving its role in maintaining mitochondrial integrity under metabolic stress.

### 3.5. Resveratrol Enhances PINK1-Mediated Mitophagy in AML-12 Cells

**Figure 5:**
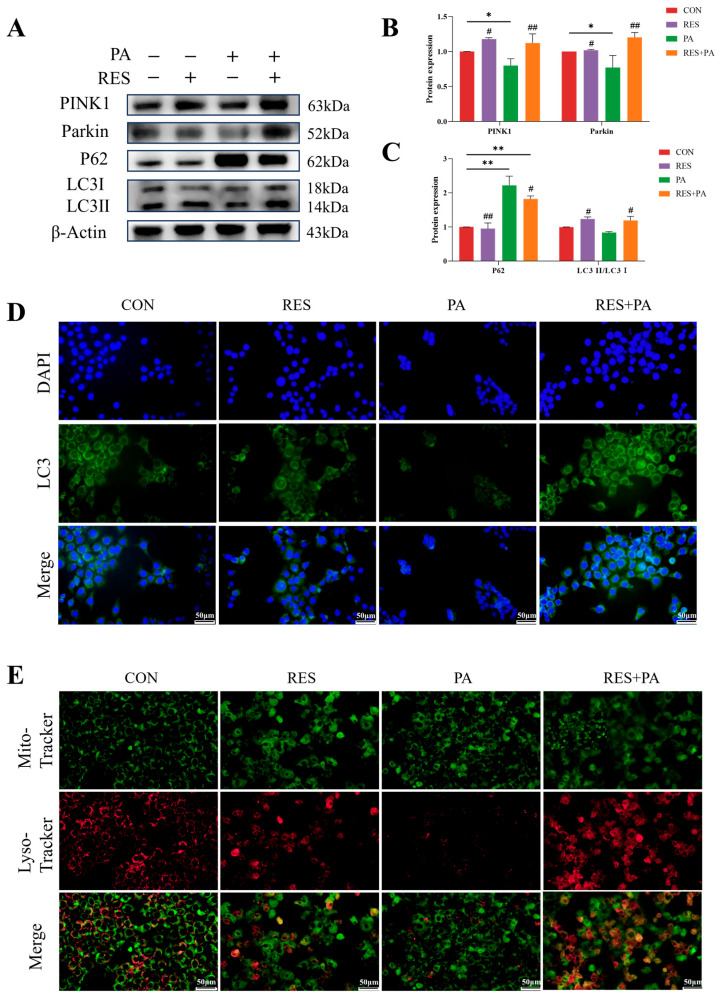
The effect of resveratrol treatment on mitophagy in PA-induced AML-12 cells. (**A**–**C**) The protein abundance of PINK1, Parkin, P62 and LC3 in AML-12 cells from different groups. (**D**) LC3 immunofluorescence staining in AML-12 cells. (**E**) MitoTracker Green and LysoTracker Red co-staining in AML-12 cells. Data are presented as mean ± SEM, * *p* < 0.05, ** *p* < 0.01 vs. CON group; ^#^ *p* < 0.05, ^##^ *p* < 0.01 vs. PA group. Scale bar, 50 μm.

Next, we studied whether resveratrol can further affect the recovery of mitophagy. As presented in Figure 5A–C, the protein expression of PINK1 and Parkin in AML-12 cells exposed to PA decreased significantly, the LC3II/LC3I ratio decreased, and the protein abundance of P62 was significantly upregulated. On the contrary, resveratrol treatment effectively alleviated these changes, showing a significant increase in PINK1, Parkin levels and LC3II/LC3I ratios, but a significant decrease in P62 expression. These observations were further verified by immunofluorescence staining using LC3-specific antibodies (Figure 5D). Furthermore, MitoTracker Green and LysoTracker Red co-staining revealed that PA exposure reduced mitochondrial–lysosome colocalization, which was markedly enhanced by RES pretreatment (Figure 5E). In short, these findings indicate that PINK1/Parkin-dependent mitophagy is damaged in PA-induced AML-12 cells. However, RES intervention effectively offsets this defect and restores mitophagy flux.

### 3.6. Suppressing Mitophagy Reversed the Protective Effect of Resveratrol on PA-Induced Mitochondrial Damage in AML-12 Cells

**Figure 6:**
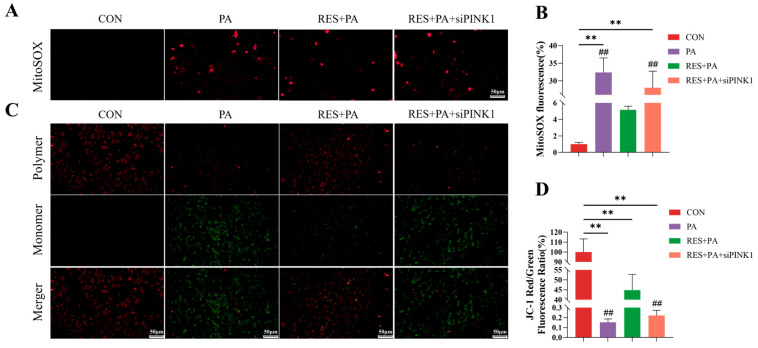
PINK1-mediated mitophagy was involved the protective effect of resveratrol on PA-induced AML-12 cell’s mitochondrial damage. (**A**,**B**) Intracellular and mitochondrial ROS generation caused by siPINK1 pretreatment in AML-12 cells. (**C**,**D**) ΔΨm changes caused by siPINK1 pretreatment in AML-12 cells. Data are presented as mean ± SEM, ** *p* < 0.01 vs. CON group; ^##^ *p* < 0.01 vs. PA + RES group. Scale bar, 50 μm.

In order to explore the mechanism of how RES affects the lipid toxicity of hepatocytes, the activity of mitophagy is inhibited by lowering the expression of PINK1 with siRNA. First of all, we studied whether PINK1-mediated mitophagy helps resveratrol to protect mitochondria in PA-exposed AML-12 cells. As depicted in Figure 6, mtROS levels were markedly higher in the RES + PA + siPINK1 group relative to the RES + PA group (Figure 6C). The mitochondrial membrane potential assay further showed that resveratrol pretreatment markedly attenuated PA-induced loss of ΔΨm, whereas PINK1 silencing considerably reduced this protective effect (Figure 6D).

### 3.7. Supressing Mitophagy Reverses the Effect of Resveratrol on Mitophagy Promotion and NLRP3 Inflammasome Inhibition

**Figure 7:**
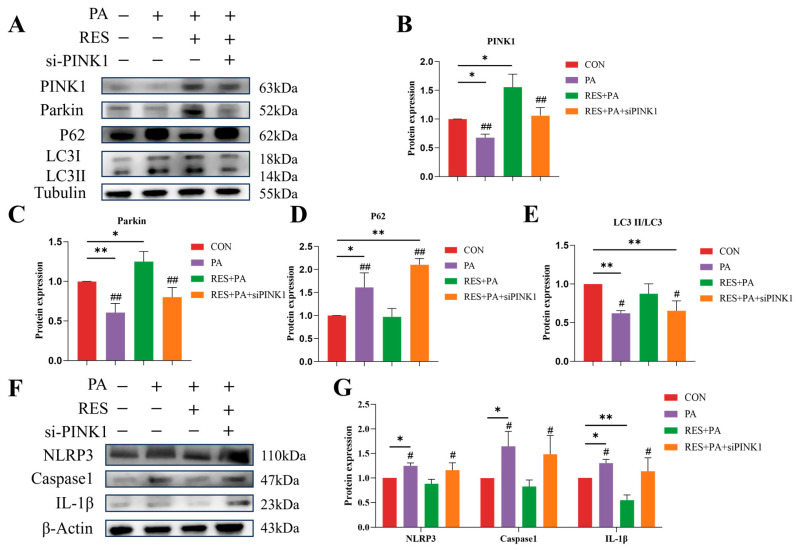
Knockdown of PINK1 suppresses resveratrol-induced hepatic mitophagy and enhances NLRP3 inflammasome activation. (**A**–**E**) The protein abundance of PINK1, Parkin, P62 and LC3 in AML-12 cells from different groups according to Western blotting; (**F**,**G**) the protein abundance of NLRP3, Caspase-1 and IL-1β in AML-12 cells from different groups according to Western blotting. Data are presented as mean ± SEM, * *p* < 0.05, ** *p* < 0.01 vs. CON group; ^#^ *p* < 0.05, ^##^ *p* < 0.01 vs. PA + RES group.

Next, we further investigate whether mitophagy is the primary pathway through which resveratrol exerts its anti-inflammatory effect. Our results demonstrated that *PINK1* knockdown attenuated the protective effects of resveratrol. Specifically, resveratrol treatment upregulated the protein expression of PINK1 and Parkin, increased the LC3II/LC3I ratio (Figure 7A–C,E), and reduced p62 accumulation (Figure 7A,E). In contrast, inhibiting mitophagy by knocking down PINK1 diminished the resveratrol-mediated mitochondrial Parkin recruitment and enhanced p62 accumulation (Figure 7A–D). Moreover, mitophagy inhibition significantly increased the protein levels of NLRP3, Caspase-1, and IL-1β in the PA + RES + siPINK group in comparison with the PA + RES group (Figure 7F,G).

### 3.8. Resveratrol Improves Hepatic Histology and Serum Biochemical Profiles in HFD-Fed Mice

**Figure 8:**
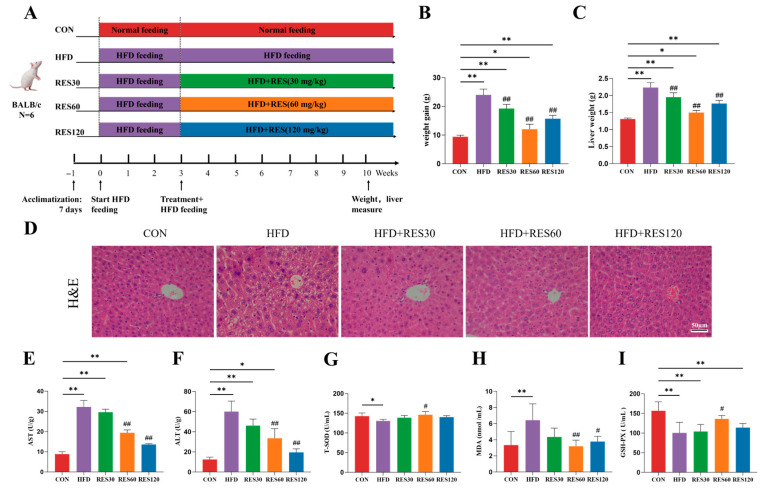
Effects of resveratrol on hepatic histomorphology and serum biochemical indicators in mice. (**A**) Experimental design. (**B**) Weight again. (**C**) Liver weight. (**D**) Representative photomicrographs of liver sections stained with H&E. (**E**) Activity of AST. (**F**) Activity of ALT. (**G**) Activity of T-S-OD. (**H**) MDA content. (**I**) Activity of GSH-Px. Data are presented as mean ± SEM, * *p* < 0.05, ** *p* < 0.01 vs. CON group; ^#^ *p* < 0.05, ^##^ *p* < 0.01 vs. HFD group. Scale bar, 50 μm.

To validate the relevance of our research findings in vivo, we used an HFD-induced fatty liver mouse model. The data in Figure 8B,C show that HFD feeding led to marked elevation of body and liver weight compared to the controls, and these increases were partially reversed by RES supplementation. Histological examination via H&E staining further revealed that RES ameliorated hepatocellular ballooning degeneration in HFD-fed mice (Figure 8D). In comparison with the control group, the HFD group showed markedly elevated serum AST and ALT activity, which was significantly attenuated with RES administration (Figure 8E,F). RES also enhances the antioxidant capacity of the liver, as evidenced by the increase in GSH-Px and T-SOD and the decrease in MDA content.

### 3.9. Resveratrol Ameliorates HFD-Induced Hepatic Steatosis and Dyslipidemia in Mice

**Figure 9:**
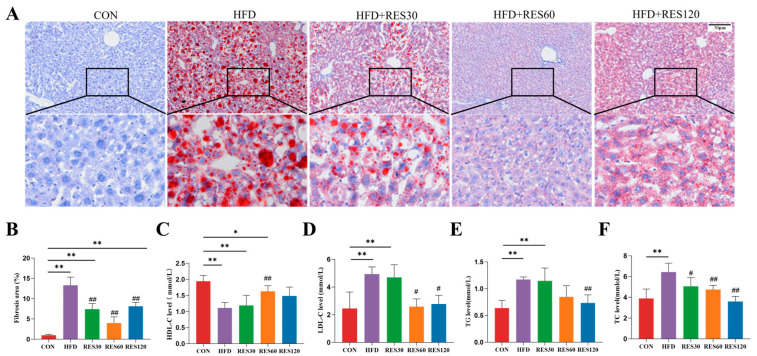
Effects of resveratrol on lipid metabolism and serum biochemical indicators in mice. (**A**) Representative photomicrographs of liver sections stained with Oil-red O (ORO). (**B**) Quantification of Oil Red O-positive areas in liver sections. (**C**) HDL-C content. (**D**) LDL-C content. (**E**) TG content. (**F**) TC content. Data are presented as mean ± SEM, * *p* < 0.05, ** *p* < 0.01 vs. CON group; ^#^ *p* < 0.05, ^##^ *p* < 0.01 vs. HFD group. Scale bar, 50 μm.

ORO staining confirmed that there was obvious lipid deposition in HFD mouse livers, and RES treatment greatly reduced this deposition (Figure 9A,B). In addition, the HFD group showed significant rises in TC, TG, and HDL-C, while LDL-C showed the opposite trend (Figure 9C–F). The application of 60 mg/kg RES leads to a significant improvement in most lipid parameters.

### 3.10. Resveratrol Suppresses NLRP3 Inflammasome Activation in the Liver of HFD-Fed Mice

**Figure 10:**
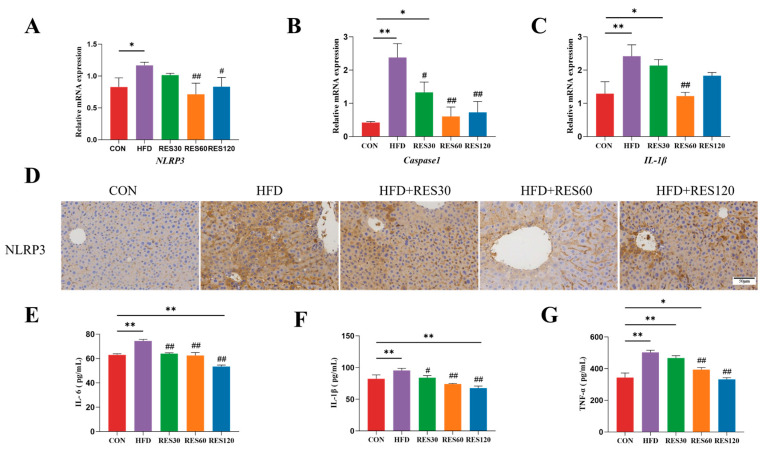
Resveratrol relieved HFD-impaired hepatic NLRP3 inflammasome activation. (**A**–**C**) mRNA abundance of *NLRP3*, *Caspase-1*, and *IL-1β* in mouse livers. (**D**) Hepatic protein abundance of NLRP3 in the livers from different groups; (**E**–**G**) the IL-6, IL-1β, and TNF-α levels in serum. Data are presented as mean ± SEM, * *p* < 0.05, ** *p* < 0.01 vs. CON group; ^#^ *p* < 0.05, ^##^ *p* < 0.01 vs. HFD group. Scale bar, 50 μm.

To further elucidate the anti-inflammatory action of RES in vivo, we evaluated its influence on hepatic tissue damage in HFD-fed mice. Following five weeks of RES administration, the increased levels of inflammatory cytokines in HFD mice were notably lowered (Figure 10). The current observations also indicate that RES administration markedly downregulated the hepatic mRNA expression of *NLRP3*, *Caspase-1*, and *IL-1β* (Figure 10A–C), and the protein levels of NLRP3, IL-6, IL-1β and TNF-α (Figure 10D–G), in HFD-fed mice. Furthermore, administration of 60 mg/kg, RES significantly attenuated inflammation-related markers compared with the HFD group.

### 3.11. Resveratrol Activates PINK1-Dependent Mitophagy in the Liver of HFD-Fed Mice

**Figure 11:**
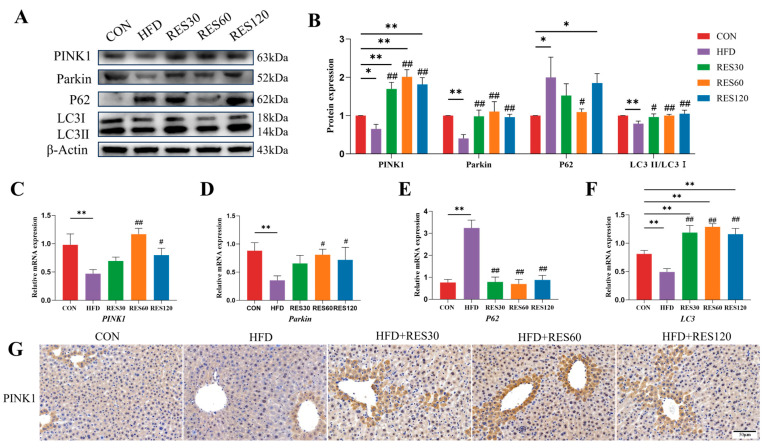
Resveratrol restores HFD-impaired hepatic mitophagy. (**A**,**B**) The protein abundance of PINK1, Parkin, P62 and LC3 in the livers from different groups according to Western blotting. (**C**–**F**) mRNA abundance of PINK1, Parkin, P62 and LC3. (**G**) Hepatic protein abundance of PINK1 in the livers from different groups. Data are presented as mean ± SEM, * *p* < 0.05, ** *p* < 0.01 vs. CON group; ^#^ *p* < 0.05, ^##^ *p* < 0.01 vs. HFD group. Scale bar, 50 μm.

Based on the function of RES in promoting mitophagy in vitro, we investigated its effects on hepatic mitophagy in a mouse fatty liver model. The results indicated that RES treatment led to a significant rise in both mRNA and protein levels of PINK1, Parkin, and the LC3-II/LC3-I ratio, while downregulating p62 expression at the transcriptional and translational levels in HFD mouse livers (Figure 11). These coordinated changes demonstrate that RES activates PINK1/Parkin-mediated mitophagy and alleviates impaired autophagic flux under HFD conditions.

## 4. Discussion

**Figure 12:**
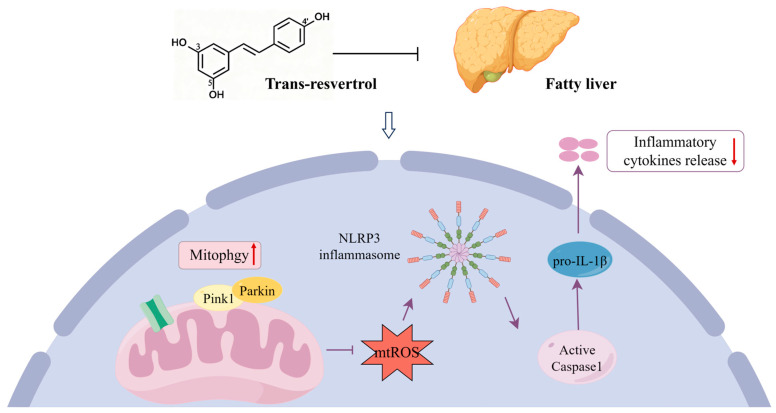
Resveratrol alleviates fatty liver disease via PINK1-mediated mitophagy and NLRP3 inflammasome suppression. Resveratrol activates PINK1-mediated mitophagy, which promotes the clearance of damaged mitochondria and reduces mtROS release. This decrease in mtROS subsequently inhibits NLRP3 inflammasome activation, leading to reduced cleavage of pro-IL-1β and Caspase-1, and ultimately attenuating the release of inflammatory cytokines. Through this pathway, resveratrol alleviates hepatic steatosis and mitigates the progression of fatty liver disease.

Fatty liver disease is a common metabolic disorder in perinatal cows, mostly caused by the excessive accumulation of liver lipids due to negative energy balance in the perinatal period [6,28]. In this study, we demonstrate that RES attenuates fatty liver injury by restoring PINK1-mediated mitophagy, thereby suppressing NLRP3 inflammasome activation (Figure 12). These findings unveiled a mechanism wherein resveratrol attenuates liver inflammation in non-alcoholic fatty liver disease through enhancement of PINK1-mediated mitophagy, suggesting its potential relevance for fatty liver disease in dairy cows.

Fatty liver development is closely related to excessive lipid accumulation in hepatocytes, which is mainly driven by the increase in circulating free fatty acid levels in the circulation, especially PA, which promotes TG deposition [29,30]. This lipid-toxic environment can aggravate liver cell injury and trigger a strong inflammatory reaction, a phenomenon verified in bovine fatty liver that manifests as an increase in liver TG content and an increase in serum ALT and AST activity, as well as up-regulation of pro-inflammatory cytokines such as IL-1β, IL-6 and TNF-α. These findings are consistent with previous reports in humans and rodents, in which lipid toxicity damages mitochondrial β-oxidation and promotes the production of reactive oxygen species, thus forming a vicious circle of metabolic dysfunction and inflammation [31,32,33,34,35].

In addition to inflammatory activation, our results also show that fatty liver is accompanied by impaired mitochondrial quality control, especially mitophagy. The expression of key mitophagy regulatory factors, including PINK1, Parkin and LC3-II/I ratio, was significantly down-regulated in the liver, while the autophagy-flow-blocking marker p62 was markedly accumulated. This indicates that lipid toxic stress not only induces inflammation, but also inhibits the clearance of damaged mitochondria. Similarly, for AML-12 hepatocytes exposed to PA, we observed a decrease in ΔΨm and an increase in mtROS levels. Together, these results strengthen the evidence that mitophagy is damaged under lipid toxicity conditions. We found that mitochondrial dysfunction and defective mitophagy promote hepatic steatosis, which is consistent with previous reports [36,37,38], and strengthens their evidence as a conservative pathological and physiological mechanism across species [39,40].

Natural polyphenol resveratrol has attracted attention for its potential to improve metabolic disorders through antioxidant and anti-inflammatory properties [41,42,43,44,45,46]. Consistent with these reports, we found that resveratrol pretreatment restored mitochondrial membrane potential, inhibited mtROS and enhanced PINK1/Parkin-dependent mitophagy in AML-12 cells. Since PINK1 is the key promoter of mitophagy, its knockdown is expected to disrupt the entire mitophagic process and exacerbate mitochondrial damage [38,47]. Importantly, the loss function experiment shows that PINK1 knock-down eliminates the protective effect of resveratrol on mitochondrial function and activation of the NLRP3 inflammasome; this indicates that resveratrol alleviates inflammation mainly through the mitophagy pathway mediated by PINK1.

To further verify the physiological relevance of these findings, we adopted a mouse model of fatty liver induced by a high-fat diet. Consistent with the findings for cultured hepatocytes, mice fed on a high-fat diet showed liver mitophagy defects and increased activation of the NLRP3 inflammasome. Resveratrol treatment in mice continuously activated PINK1/Parkin pathway, promoted the transformation of LC3-II, reduced p62 accumulation and inhibited the expression of NLRP3, Caspase-1 and IL-1β. These changes were accompanied by significant improvements in liver steatosis, serum lipid spectrum and antioxidant capacity. The liver-protective and autophagy-regulating effects of resveratrol observed in our model are consistent with its previously reported efficacy in relieving hepatic steatosis and lipid metabolism disorders across a variety of species and disease backgrounds [48,49]. The consistency of these results between species and experiments emphasizes that resveratrol plays a liver-protective role through a conservative mechanism involved in restoring mitophagy and subsequently reducing NLRP3-driven inflammation.

Although previous studies on fatty liver-related inflammation mainly focus on cytokine spectrum analysis and oxidative stress [50,51,52], emerging evidence shows that autophagy damage exists in this context [53,54,55]. Extended rodent and human studies emphasize that mitophagy is a promising therapeutic target for metabolic diseases, and our findings provide direct evidence that resveratrol breaks the vicious circle between lipid toxicity, mitochondrial damage and inflammation by activating PINK1-dependent mitophagy. It should be noted that the limitation of this study is that while the therapeutic effect of resveratrol has been validated in rodent models and in vitro experiments, it still needs to be further verified in dairy cows. Several limitations of the present work merit consideration. First, although pathway alterations were documented in bovine fatty liver specimens, the therapeutic benefits of resveratrol have thus far been demonstrated solely in rodent models and in vitro experiments. Therefore, before any clinical application in dairy cows can be considered, further studies are essential to establish its pharmacokinetic profile, optimal dosage, and therapeutic efficacy in the target species. Secondly, although the use of murine AML-12 cells allowed mechanistic exploration of the PINK1/Parkin pathway, potential interspecies differences in mitochondrial and inflammatory signaling warrant consideration. Validation using primary bovine hepatocytes represents an important direction for future research to establish the clinical relevance of these findings for dairy cattle. Unlike prior work focusing on PI3K/AKT/mTOR signaling in NAFLD [56], we demonstrate that resveratrol alleviates liver inflammation through PINK1-dependent mitophagy, identifying a new mechanistic pathway. Despite these limitations, our results indicate that resveratrol is worthy of further investigation as a potential nutritional strategy to manage fatty liver in transition dairy cows by modulating mitochondrial quality control and inflammatory pathways, although it still needs to be validated in cow-specific studies.

## 5. Conclusions

In summary, our research indicates that resveratrol improves fatty liver disease by enhancing mitophagy, coupled with inhibiting NLRP3-driven inflammation. These findings not only provide new molecular insights into the pathogenesis of bovine fatty liver, but also pave the way for resveratrol-based interventions, offering a feasible and active strategy for controlling fatty liver disease in dairy cows during the transition period.

## Data Availability

The data supporting this study’s findings are available from the corresponding author upon reasonable request.

## Supplementary Materials

The following supporting information can be downloaded at: https://www.mdpi.com/article/10.3390/ani16071022/s1, Table S1. Primer sequences used in real-time quantitative PCR assay. Figure S1. Screening of palmitic acid (PA) and resveratrol (RES) concentrations in AML-12 cells. (A) Viability of AML-12 cells treated with various concentrations (0, 200, 400, 600, 800 μM) of PA for 24 h, as determined by CCK-8 assay. (B,C) mRNA expression levels of IL-1β and TNF-α in AML-12 cells after treatment with various concentrations PA for 24 h. (D) Viability of AML-12 cells treated with various concentrations (0, 15, 30, 60, 120 μM) of RES for 24 h. (E,F) mRNA expression levels of IL-1β and TNF-α in cells treated with RES. Data are presented as mean ± SEM. Statistical differences were assessed by one-way ANOVA with subsequent Tukey’s honestly significant difference test. * *p* < 0.05, ** *p* < 0.01 vs. PA (0 μM) +RES (0 μM) group; ^#^ *p* < 0.05, ^##^ *p* < 0.01 vs. PA (600 μM) + RES (0 μM) group.
