# Supplementary material for: Resveratrol Attenuates Liver Inflammation in Non-Alcoholic Fatty Liver Disease by Activating PINK1-Mediated Mitophagy
Source: Animals (Basel). 2026 Mar 27;16(7):1022. doi: 10.3390/ani16071022 (PMC13072143; doi:10.3390/ani16071022)

Supplementary Materials

Table S1 Primer sequences used in real-time quantitative PCR assay.

Gene	Forward primer (5’-3’)	Reverse primer (5’-3’)
PINK1	CCCCAGTGCGGTAATTGACT	CTAGAAGATGCTCGCCCCAG
Parkin	GCAAACAAGCAACCCTCACC	CAGTGGAGATGAGGCCGAGT
IC3B	AAGAGTGGAAGATGTCCGGC	GGCTCCTATGGGGTTAGGGT
P62	ACTGCTCAGGAGGAGACGAT	AACCCATGGACAGCATCTGG
NLRP3	TATCCACTGCCGAGAGGTGA	TCTTGCACACTGGTGGGTTT
IL-1β	ACTCAACTGTGAAATGCCACC	TGATACTGCCTGCCTGAAGC
IL-6	AGCCAGAGTCCTTCAGAGAGA	GCCACTCCTTCTGTGACTCC
IL-18	ACTTTGGCCGACTTCACTGT	CAGTCTGGTCTGGGGTTCAC
β-actin	ATATCGCTGCGCTGGTCG	TTCCCACCATCACACCCTGG

Figure. S1. Screening of palmitic acid (PA) and resveratrol (RES) concentrations in AML-12 cells. (A) Viability of AML-12 cells treated with various concentrations (0, 200, 400, 600, 800 μM) of PA for 24 h, as determined by CCK-8 assay. (B- C) mRNA expression levels of IL-1β and TNF-α in AML-12 cells after treatment with various concentrations PA for 24 h. (D) Viability of AML-12 cells treated with various concentrations(0, 15, 30, 60, 120 μM) of RES for 24 h. (E-F) mRNA expression levels of IL-1β and TNF-α in cells treated with RES. Data are presented as mean ± SEM. Statistical differences were assessed by one-way ANOVA with subsequent Tukey's honestly significant difference test. * $p < 0.05$, ** $p < 0.01$ vs PA (0μM) +RES (0μM) group; $^{\#}p < 0.05$, $^{\#\#}p < 0.01$ vs PA (600μM) +RES (0μM) group.

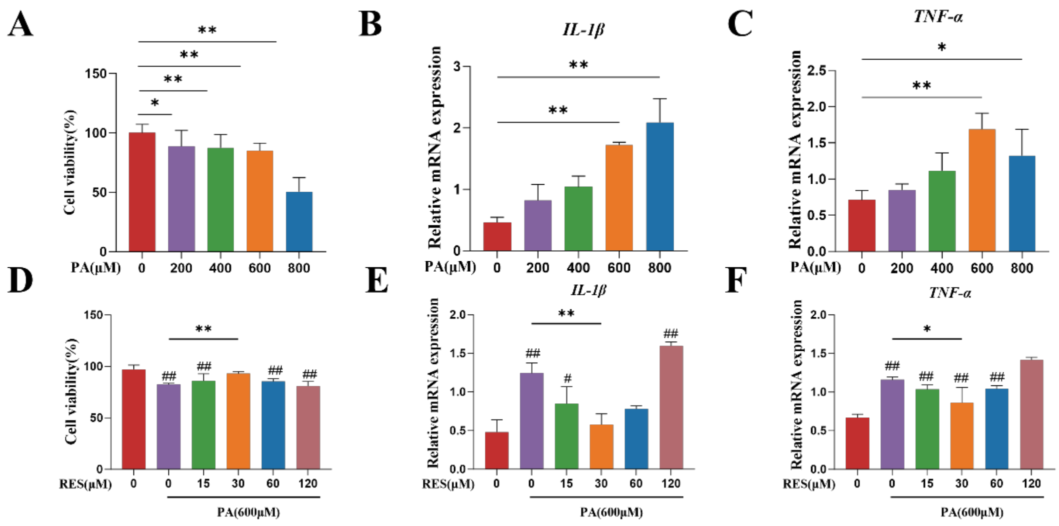

Supplement: Supplementary file 1 [file animals-16-01022-s001.zip › animals-4195744-supplementary.pdf]
